# Chemical Composition and Antioxidant Activity of *Heracleum sprengelianum* (Wight and Arnott) Essential Oils Growing Wild in Peninsular India

**Published:** 2011

**Authors:** Subbiah Karuppusamy, G Muthuraja

**Affiliations:** *Department of Botany, Botanical Research Center, Madura College, Madurai–625 011, Tamil Nadu, India.*

**Keywords:** *Heracleum sprengelianum*, Essential oils, GC-MS, Antioxidant activity, Thiobarbituric acid reactive substances assay

## Abstract

The essential oils, isolated by hydrodistillation from the leaves, seeds and rhizomes of *Heracleum sprengelianum *(Wight and Arnott), collected from the Western Ghats of Peninsula India, were analyzed by gas chromatography (GC) and gas chromatography coupled to mass spectrometry (GC–MS). The antioxidant property of these oils was tested, with and without peroxidation inducer, through the egg yolk-based Thiobarbituric Acid Reactive Substances assay (TBARS assay) and in the concentrations of 50, 100, 250 and 500 mg/L. *β*-Pinene, 1,8-Cineole, *β*-Phellandrene and *ρ*-Cymen-8-ol were the main components of *H. sprengelianum *leaves, seeds and rhizomes essential oils. The oils demonstrated the antioxidant capacity in the absence of radical inducer 2, 20-azobis-(2-amidinopropane) dihydrochloride (ABAP), mainly that of *H. sprengelianum *at 250 and 500 mg/L, comparable in some cases to that of *α*-tocopherol and butylated hydroxytoluene (BHT). The presence of ABAP diminished the antioxidant ability of all tested essential oils, leaf oils of *H. sprengelianum *still showing the highest antioxidant capacity at 500 mg/L. At 250 and 500 mg/L for BHA, and 500 mg/L for *α*-tocopherol, the antioxidant capacity significantly increased in the presence of ABAP.

## Introduction

Free radicals are potentially important in a number of ailments states that can have severe effects on the cardiovascular system, either through lipid peroxidation or vasoconstriction (1). Although the antioxidant defense systems include both endogenously and exogenously derived compounds, dietary plant-based antioxidants have recently received a great attention (2). Hence, many studies have been performed to identify antioxidant compounds with pharmacological activity and a limited toxicity from medicinal plants.

Nowadays, there is a great world-wide interest in finding new and safe antioxidants from natural sources to prevent oxidative deterioration of foods and to minimize the oxidative damages in living cells. Traditionally, chemically synthesized compounds, such as butylated hydroxy-anisole (BHA) and butylated hydroxytoluene (BHT), are used as antioxidants in oil products. However, some of these compounds have been questioned for their safety (3). The use of BHA and BTH is proved to be carcinogenetic (4). Therefore, there is an increasing interest in the antioxidative activity of natural compounds (5). Higher and aromatic plants have traditionally been used in folk medicine as well as to extend the shelf life of foods (6). Most of their properties are due to essential oils produced by their secondary metabolism (7). Several of them are qualified as antioxidant and are proposed to replace synthetic antioxidants used in food industry where they do not affect the organoleptic characteristics of the final product. Furthermore, numerous scientific reports have highlighted an important antioxidant activity of essential oils (8-10). These biological activities depend on the chemical composition (11) which varies according to the geographical origin, the environmental and agronomic conditions, the stage of development of the plant material and the extraction method (12). Therefore, the evaluation of the biological activity of an essential oil should be supplemented with the determination of its chemical composition.

*Heracleum *L. (Apiaceae) includes more than 70 species in the world. There are 15 species in India, 5 of which are endemic to Peninsular India (13). *Heracleum sprengelianum *is growing wildly in grasslands of Western Ghats at a height of 1500 m. The rhizome and seeds are used as folk medicines. They are reported to be effective in curing indigestion, sunburn, skin diseases and external tumors (14). Many studies have reported the biological activities and essential oil composition of the genus *Heracleum *(15-19). As part of our on-going studies on the genus *Heracleum *from Western Ghats of India, we are now reporting the composition of the volatile oils of the rhizome, leaves and seeds of *H. sprengelianum *for the first time, together with its antioxidant properties. In this context, ethnopharmacology plays a significant role in search of interesting and therapeutically useful plants. In order to add to the knowledge of plants from Western Ghats of India, in the present study, *H. sprengelianum *(Apiaceae) essential oils were screened to determine their free radical scavenging and antioxidant activities.

## Experimental

*Plant material*

The leaves, seeds and rhizomes of *Heracleum sprengelianum *(Wight and Arnott) were collected, during December 2009, from the Nilgiri hills, Western Ghats of Peninsular India. The identification of the specimens authenticated with comparison of herbarium sheets deposited in Botanical Survey of India, Southern Circle, Coimbatore, Tamil Nadu. The voucher specimens (voucher no. *SK and GM *1427, 1462) have preserved in Department of Botany, The Madura College, Madurai, Tamil Nadu.

*Isolation procedure*

The essential oils of leaves, mature seeds and rhizomes of *H. sprengelianum *were isolated from fresh plant material (200 g) by hydrodistillation, for 4 h, using a Clevenger-type apparatus.

*Gas chromatography*

The *Gas Chromatography *(GC) analysis was carried out with a Hewlett-Packard HP6890, equipped with a HP-Innowax silica capillary column (60 m x 0.25 mm, film thickness 0.25 μm) and a flame ionization detector. Nitrogen was used as carrier gas with a flow rate of 0.8 mL/min. Injector and detector temperatures were both set at 250°C. Column temperature was programmed to 60°C for 10 min, gradually increased to 220°C at 4°C /min , held for 10 min and then increased to 240°C at 10°C /min. Split ratio was 50:1 and one μL of sample (dissolved in hexane as 20% v/v) was injected into the system.

*Gas chromatography-mass spectrometry*

The *Gas chromatography-mass spectrometry *(GC-MS) analysis of the oil was carried on an Agilent 6890N Network GC system combined with Agilent 5973 Network Mass Selective Detector (GC-MS). The employed capillary column was an Agilent 19091N-136 (HP Innowax Capillary; 60.0 m × 0.25 mm × 0.25 μm). Helium was used as carrier gas at a flow rate of 1.0 mL/min with 1 μL injection volume. Samples were analyzed with the column held initially 60°C after injection with 10 min hold time, then increased to 220°C with 4°C /min heating ramp and kept at 220°C for 10 min. Then, the final temperature was increased to 240°C with 1°C /min heating ramp. The injection was performed in split mode (50:1). Detector and injector temperatures were 230°C and 280°C, respectively. Run time was 80 min. MS scan range was (*m/z*): 35-450 atomic mass units (AMU) under electron impact (EI) ionization (70 eV).

*Identification of volatile oil components*

The components were identified by comparing their relative retention times with those of authentic samples and mass spectra with the data from the Baser library of essential oil constituents as well as Wiley and Nist Library. Relative content of % components were determined with the area under peaks using Agilent software. The results are expressed as an average of three determinations in all cases. GC and GC/MS analysis were both conducted at the Sophisticated Analytical Instrument Facility (SAIF), Central Drug Research Institute, Lucknow, India.

*Antioxidant activity measurement*

Two sets of experiments based on a modified TBARS assay were used to measure the antioxidant ability of the sample (essential oils or tested substances), with and without a lipid peroxidation inducer. In both cases, egg-yolk homogenates were used as a lipid-rich media (20); *i.e*., an aliquot of yolk material was made up to a concentration of 10% (w/v) in KCl (1.15%, w/v). The yolk was then homogenized for 30 sec, followed by ultrasonication for a further 5 min. For set 1 of TBARS assay, 500 μL of 10% (w/v) homogenate and different concentrations of sample (50, 100, 250 and 500 mg/L), solubilized in methanol, were added to a test tube and made up to 1 mL with distilled water, followed by adding 1.5 mL of 20% acetic acid (pH 3.5) and 1.5 mL of 0.8% (w/v) TBA in 1.1% (w/v) sodium dodecyl sulfate (SDS). Each essential oil and tested substance was assayed at the concentrations of 50, 100, 250 and 500 mg/L. This mixture was stirred in a vortex, and heated at 95°C for 60 min. After cooling at room temperature, 5 mL butan-1-ol was added to each tube, stirred and centrifuged at 3000 rpm for 10 min. The absorbance of the supernatant was measured at 532 nm using a spectrophotometer Schimadzu 160-UV. All the values were expressed as antioxidant index (AI%), whereby the control is completely peroxidized and each oil and tested substance demonstrated a comparative percentage of antioxidant protection. The AI% was calculated using the following formula: 

(1 – *t */ C) × 100

In this formula, C is the absorbance value of the fully oxidized control and *t *is the absorbance of test sample (21). For set 2 of the TBARS assay, 50 μL of 2, 20-azobis-(2-amidinopropane) dihydrochloride (ABAP) (0.07M) was added to induce the lipid peroxidation, soon after the addition of sample, the remaining procedure was as reported above.

*Statistical analysis*

The analytical values of the antioxidant activity measurement represent means of three replicates, done in two different experiments. The obtained data was subjected to the one-way analysis of variance and Tukey test analysis. Significance was assumed at p < 0.05.

## Results and Discussion

The oil yields obtained from the different parts of *H. sprengelianum*, varied considerably (Table 1).

**Table 1:** Chemical composition of the leaves, seeds and rhizome oils of *Heracleum sprengelianum*

**Compounds**	**RI**	**Leaves (%)**	**Seeds (%)**	**Rhizome (%)**
**1-Hexanol**	867	0.08	0.02	0.06
**Heptanal**	904	0.21	0.09	--
***α*** **-Thujene**	929	0.21	0.18	0.22
***α*** **-Pinene**	952	1.68	1.27	0.84
**1-Heptanol**	965	1.26	1.22	1.82
**Sabinene**	972	8.74	--	2.12
***Β*** **-Pinene**	976	16.18	22.26	21.84
**Myrcene**	985	0.84	0.15	0.14
***α*** **-Phellandrene**	995	--	1.05	0.82
***n*** **-Octanal**	1003	1.02	1.00	--
**1,8-Cineole**	1006	21.20	20.32	23.10
**Limonene**	1026	3.16	2.12	5.24
***Β*** **-Phellandrene**	1028	11.35	12.38	15.19
***cis*** **-Ocimene**	1034	0.18	---	0.10
***Trans-Sabinene***				
**hydrate**	1039	0.29	0.21	0.16
**1-Octanol**	1067	1.12	1.85	2.46
***α*** **- Terpinolene**	1086	1.57	1.25	0.48
**Camphor**	1095	0.49	0.15	1.20
**Linalool**	1098	0.28	0.52	0.15
***trans*** **-Pinocarveol**	1121	--	1.21	0.46
**Nerol oxide**	1128	0.28	0.18	0.29
**Verbenol**	1141	0.32	0.51	1.48
**Terpinen-4-ol**	1149	1.39	---	1.04
***ρ*** **-Cymen-8-ol**	1183	6.64	4.40	2.22
**trans-Carveol**	1187	0.11	0.10	--
***α*** **-Terpineol**	1189	0.13	1.20	1.21
**Myrtenol**	1193	0.62	--	0.30
**Nerol**	1206	0.10	1.38	--
***1-Ethyl2,4-dimethyl***				
**Benzene**	1222	0.16	0.22	--
**Phellandral**	1227	0.12	--	0.25
**Geraniol**	1238	1.98	1.42	1.39
**trans-Decanol**	1258	0.76	0.24	0.52
**trans-Anethole**		1281	1.25	--
**Thymol**	1290	0.41	--	0.18
**2-Dodecanol**	1298	--	0.76	0.84
**Geranylacetate**		1370	1.68	1.39
***δ*** **-Selinene**	1381	0.36	0.29	--
***β*** **-Caryophyllene**	1429	2.62	7.20	1.30
***α*** **-Humulene**	1447	0.32	--	0.12
**Myristicin**	1514	0.26	0.14	--
**Kessane**	1520	1.66	1.25	0.28
**Hedycariol**	1537	0.25	0.14	2.42
**Nerolidol**	1552	0.26	0.14	1.39
**Spathulenol**	1564	1.29	1.52	0.48
**Calarene**	1571	1.52	1.39	0.82
**Viridiflorol**	1583	2.01	2.48	1.53
**Valerenol**	1660	0.10	0.35	--
**Identified components (%)**		99.46%	93.95%	97.62%
**Grouped components**				
**Monoterpene hydrocarbons**		91.5%	87.7%	90.0%
**Oxygenated monoterpenes**		2.8%	2.1%	1.4%
**Sesquiterpene hydrocarbons**		4.8%	1.8%	4.8%
**Oxygenated sesquiterpenes**		0.2%	1.3%	1.5%
**Total identified **		99.3%	93.9%	97.6%

 The highest oil yield was obtained from leaves and seeds (1.3% and 1.1% v/w), while the lowest one was obtained from rhizome (0.7%). These yields were relatively lower than the average oil yields reported in related species of *H. candolleanum *from Western Ghats of India (22). The different harvesting period of the samples could partly be responsible for these differences since both the oil yield and the proportions of the several constituents of essential oil may vary greatly according to the developmental phase of the plant (23).

A total of forty-six components could be identified, representing 99% of the total oils from leaves, seeds and rhizomes, which are listed in Table 1. Although monoterpenes were dominant in all oils (leaves 91.5%; seeds 87.7% and rhizome 90% respectively), the importance of the oxygen-containing or monoterpene hydrocarbons varied. The essential oils of *H. sprengelianum *leaves were dominated by monoterpenes (91.5%), whereas in seed oils, monoterpenes (87%) and the rhizome oils, monoterpene hydrocarbons (90%) were present in more approximate amounts.

1,8-Cineole was the dominant component in rhizome (23.10%) and leaves (21.20%) of *H. sprengelianum *essential oil, which is in accordance with the report by Saraswathy and Sasikala (22) for the *Heracleum *spp. oils, though different from most of the Indian species of *Heracleum *chemotypes. *β*-Phellandrene (11.35%) (*β*-Pinene (16.18%), 1,8-cineole (21.20%), Sabinene (8.74%), *ρ*-Cymene-8-ol (6.64%), and *β*-Caryophyllene (2.62%) dominated components in leaves of *Heracleum sprengelianum *oil, showing some similarities to some previously studied populations of related species of *H. candolleanum *and *H. concanense *(25, 26). *β*-Pinene and 1,8-Cineole were the main components in all the parts of *Heracleum *oils, which is also in agreement with previous reports for other species (27).

All the essential oils showed some antioxidant capacity, in the absence of the radical inducer, increasing over the concentration range tested (Table 2).

**Table 2:** Antioxidant index (%) of the essential oils, *α*-tocopherol, BHT and BHA, in different concentrations (mg/L), using TBARS assay without ABAP

**Oil/Substance**	**Concentration (Mean ± SE (mg/L))**			
	50	100	250	500
***α*** **-Tocopherol**	68.2 ± 3.2	70.2 ± 4.0	74.0 ± 1.5	74.2 ± 1.4
**BHA**	60.3 ± 3.7	64.9 ± 4.2	64.0 ± 1.5	64.3 ± 1.4
**BHT**	72.5 ± 3.2	75.3 ± 4.0	81.2 ± 1.2	82.9 ± 1.4
**Leaf oil**	25.4 ± 3.5	29.4 ± 4.0	36.8 ± 1.5	38.5 ± 1.4
**Seed oil**	20.2 ± 3.4	21.9 ± 3.8	32.1 ± 1.4	34.2 ± 1.4
**Rhizome oil**	18.6 ± 3.2	20.1 ± 3.2	28.4 ± 1.4	30.1 ± 1.4

SE: Standard Error. Means with different superscript letters are significantly different (p < 0.05).

*H. sprengelianum *leaf oils showed the highest antioxidant index at 250 and 500 mg/L, comparable to or higher than that of *α*-tocopherol and BHA, respectively, but still lower than that of BHT, at the same concentrations. The essential oils of *H. sprengelianum *rhizome showed much lower antioxidant indices than that of seed oils. The decrease in concentration of the oils produced a drastic reduction in their activity and the oils were scarcely active at the lowest concentration. (50 mg/L).

With the exception of the oils of *H. sprengelianum *and in comparison with the previous experiment, the remaining essential oils showed a decrease in their antioxidant capacities in the presence of the radical inducer, ABAP (Table 3). Again, the highest degree of the tested oils activity was detected at 500 mg/L, for the oil of *H. sprengelianum *leaves, but this was still lower than that of the synthetic antioxidants and *α*-tocopherol. Negative antioxidant activity was observed in low concentrations of leaf and rhizome oils.

**Table 3:** Antioxidant index (%) of the essential oils, *α*-tocopherol, BHT and BHA, in different concentrations (mg/L), using TBARS assay with ABAP

**Oil/Substance**	**Concentration (mean ± SE (mg/L))**			
	50	100	250	500
*α*-Tocopherol	48.5 ± 5.1	65.8 ± 6.5	75.2 ± 4.0	81.2 ± 3.5
BHA	53.2 ± 5.1	62.8 ± 6.5	73.2 ± 4.0	81.0 ± 3.5
BHT	40.8 ± 5.1	50.2 ± 6.5	64.8 ± 4.0	68.6 ± 3.5
Leaf oil	-34.5 ± 5.1	1.2 ± 6.5	21.2 ± 4.0	40.8 ± 3.5
Seed oil	6.4 ± 5.1	15.4 ± 6.5	32.5 ± 4.0	36.4 ± 3.5
Rhizome oil	-18.4 ± 5.1	-12.5 ± 6.5	-8.4 ± 4.0	15.3 ± 3.5

SE: Standard Error; Means with different superscript letters are significantly different (p < 0.05).

The different behaviour of the synthetic antioxidants and *α*-tocopherol in the absence/presence of ABAP suggests that BHA and *α*-tocopherol are able to operate as good antioxidants when the levels of peroxyl radicals are relatively high, whereas BHT and the essential oils tested do not seem to possess such ability. According to some authors (28, 29), this behaviour may have a structural explanation. BHA and *α*-tocopherol have a lower steric hindrance around the phenol function compared to BHT, which possesses two tetra-butyl groups in the ortho positions. BHT would therefore, prevent hydrogen bonding occurring in water solutions. The highest hydrogen bonding of water molecules to the phenol function in *α*-tocopherol and BHA, due to their reduced steric hindrance, may make them less reactive toward radicals in the reaction system, decreasing the antioxidant capacity. Consequently, the capacity of BHA and *α*-tocopherol to sequester peroxyl radicals increases with the increase in their production. Further work will be required to fully characterize the potential of essential oils as protectors of highly unsaturated lipids. The fact that *H. sprengelianum *oils, which have no phenolic compounds, showed the highest antioxidant activity demonstrates that the presence of this type of compound is not obligatory for the antioxidant activity.
